# Exploring holistic care needs in paediatric and teenage molecular radiotherapy: a parent and carer perspective

**DOI:** 10.1007/s00520-026-11214-8

**Published:** 2026-09-22

**Authors:** Connie Peet, Sara Stoneham, Jenny Gains, Charlotte Leger, Mark N. Gaze

**Affiliations:** 1 Department of Radiotherapy, University College London Hospitals NHS Foundation Trust, London, UK; 2 Department of Paediatric and Adolescent Oncology, University College London Hospitals NHS Foundation Trust, London, UK; 3 Department of Oncology, University College London Hospitals NHS Foundation Trust, 250 Euston Road, London, NW1 2PG UK; 4 Department of Paediatric Nursing, University College London Hospitals NHS Foundation Trust, London, UK

**Keywords:** Holistic needs assessment, Multidisciplinary team, Children, Teenagers, Molecular radiotherapy

## Abstract

**Purpose:**

Molecular radiotherapy (MRT) for children and young people presents unique challenges distinct from other cancer treatments. It requires specialised inpatient care in paediatric settings, strict radiation safety protocols, and is typically available only at selected centres, making access uneven across regions. Despite the complexity of this treatment, there is a notable lack of research into the holistic needs of parents and caregivers of paediatric patients undergoing MRT. Understanding and addressing these needs could help enhance future care, facilitate earlier interventions, and improve care planning for both patients and their families.

**Methods:**

A prospective, systematic assessment of holistic needs was conducted for paediatric and adolescent patients undergoing their first cycle of MRT at a single tertiary referral centre between 2018 and 2022. The evaluation focused on physical, emotional, social, and practical concerns impacting the caregivers.

**Results:**

Fifty patients were included in the study, treated with [^131^I]sodium iodide, [^131^I]meta-iodobenzylguanidine (mIBG), or [^177^Lu]lutetium DOTATATE. The median patient age was 13 years. Thematic analysis identified several key areas of concern: limited access to psychosocial support services, financial burdens due to travel and time off work, and MRT-specific challenges including enforced isolation and separation from family members during treatment.

**Conclusion:**

Holistic needs assessments are vital in identifying and addressing unmet needs in MRT. A coordinated, family-centred, multidisciplinary approach is essential. Future improvements should include enhanced training for healthcare professionals, use of virtual peer support, support for education continuity, and better integration of local resources to support families throughout the treatment journey.

**Supplementary Information:**

The online version contains supplementary material available at https://doi.org/10.1007/s00520-026-11214-8.

## Introduction

One structured method for the collation of clinically relevant information about an individual’s requirements across various areas including psychological, social, physical and spiritual care, is a holistic needs assessment (HNA) [1]. An accurately completed HNA will facilitate personalised patient-centred care in a comprehensive way, which is especially valuable in oncology where the prognosis may be uncertain [2] In paediatric practice it includes the needs and values of the patient’s family as a whole.

A variety of HNA tools is available, in electronic or paper questionnaire format, to be completed either remotely or during a consultation with a healthcare professional. In general, HNA has been found to be: feasible to collect, acceptable to patients and families, of value in improving communication between patients and clinicians, and useful in promoting personalised care in the most appropriate way [3].

Molecular radiotherapy (MRT) involves the oral or intravenous administration of radioactive drugs which biologically target tumour cells. Radiation protection precautions are required due to potential environmental contamination and high external dose rates during treatment [4].

The various MRT treatments used, their indications, activities and typical lengths of stay are shown in Table 1.

**Table 1:** MRT treatments used, their indications, activities and typical lengths of stay

Molecular radiotherapy treatment type	Indication	Usual activity administered	Typical length of inpatient stay
[^131^I] sodium iodide	Differentiated thyroid cancer (papillary or follicular type)	1.1 GBq for ablation 3.0 GBq for ablation (in high-risk cases) 5.5 GBq for therapy courses in metastatic cases	2–3 days for ablation 4–7 days for therapy Longer if unfavourable home circumstances preclude early discharge
[^131^I] meta iodobenzylguanidine	Relapsed or refractory high-risk neuroblastoma	Two administrations two weeks apart 1. Weight-based 444 MBq per kg 2. Top up to achieve whole body absorbed radiation dose of 4 Gy or Two administrations two weeks apart 1. Weight-based 222 MBq per kg 2. Top up to achieve whole body absorbed radiation dose of 2 Gy	28–35 days depending on clearance of radiation and home circumstances
[^131^I] meta iodobenzylguanidine	Metastatic phaeochromocytoma and paraganglioma	7.4 or 11.1 GBq depending on the size of the child	7–14 days depending on clearance of radiation and home circumstances
[^177^Lu] Lutetium DOTATATE	Relapsed or refractory high-risk neuroblastoma	Weight-based 75–100 MBq per kg	3 days
[^177^Lu] Lutetium DOTATATE	Metastatic phaeochromocytoma and paraganglioma	7.4 GBq	3 days

**Fig. 1:**
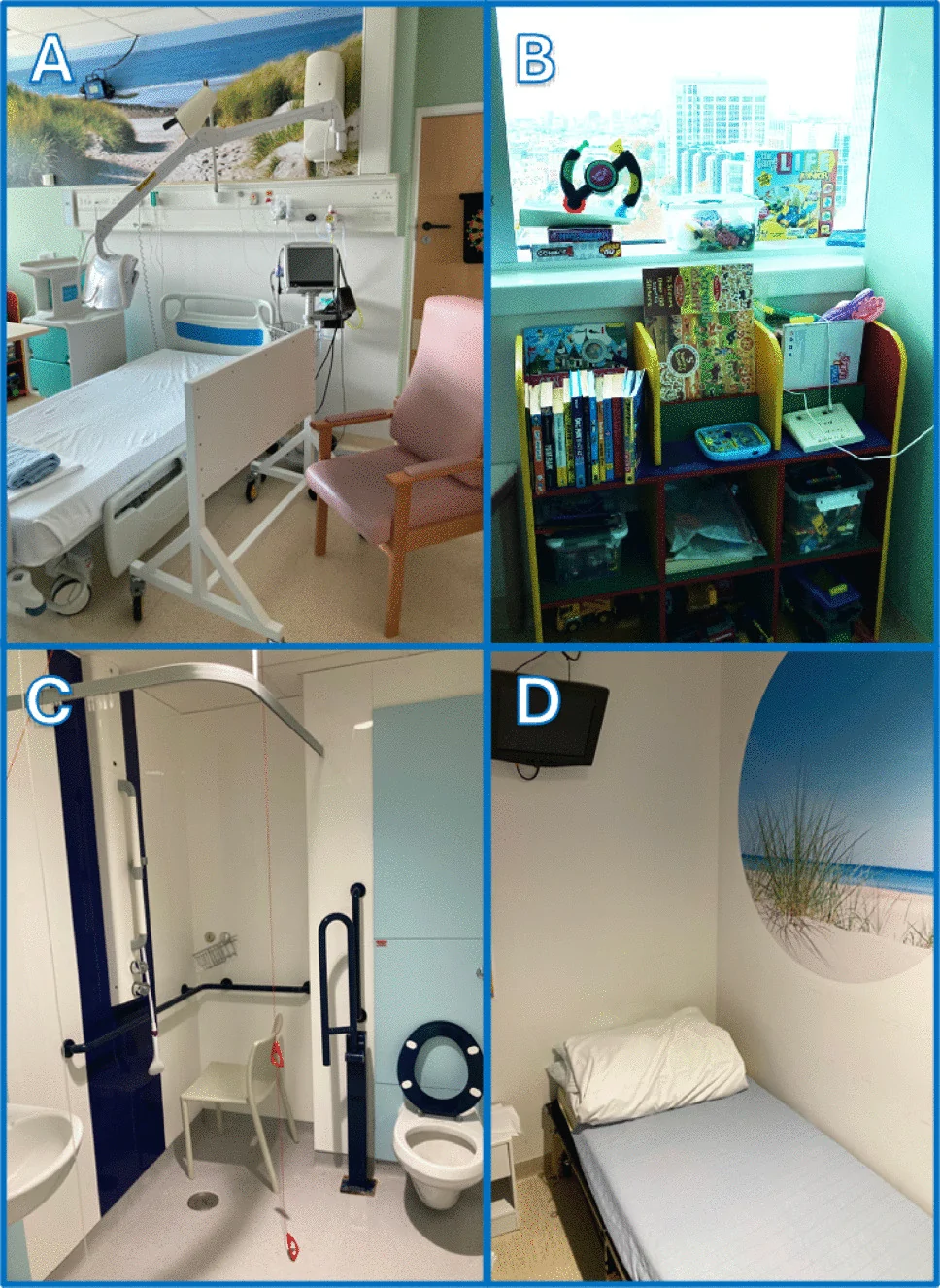
To illustrate aspects of the patient isolation facilities for paediatric molecular radiotherapy. **A** The patient’s bed, and a chair for the comforter and carer to use with an intervening lead radiation shield on wheels. Note also physiological measurement instruments and entertainment screen for the patient. **B** Large picture window allowing a good view of the outside world, and shelving with a range of age-appropriate books, games and other entertainments for the child. **C** En suite facilities comprising shower, lavatory and wash-hand basin for the child. **D** Adjacent cubicle for the comforter and carer to sleep in, protected from radiation from the treatment room but allowing immediate access to the child if needed at night, with closed circuit television monitor to observe the child

MRT is unique, and differs from other cancer treatments due to the specialist inpatient suites needed for radiation safety, and stringent guidelines around treatment [5]. Patients must stay in isolation until their level of radioactivity has diminished to an acceptable level, to minimise radiation exposure to staff and other members of the public, as set out by the Ionising Radiation Regulations 2017 (IRR17) legislation (Fig. 1) [6]. Parents or other consenting non-pregnant adults may be legally designated as “comforters and carers”. They must be informed about the precautions they must take to keep their personal radiation exposure as low as reasonably achievable. This includes the use of personal protective equipment (aprons, gloves and overshoes) to reduce the risk of contamination by radioactive body fluids, and minimising the time in close contact with the patient, maximising the distance from the patient, and the use of barriers to limit direct irradiation. Comforter and carer radiation doses must be monitored [7]. The psychological impact of radiation protection requirements has been recognised. Isolation can negatively impact patients’ and their families’ psychological well-being [8]. Radiation contamination risks associated with MRT limit personal possessions such as clothing and “home comforts” being brought into the hospital, which is an additional challenge for patients and their families [9].

MRT services for children and young people require co-location with round the clock paediatric nursing and medical care, as well as specialised nuclear medicine facilities. As most hospitals cannot offer this, services are centralised to only a few locations in the United Kingdom. This presents significant challenges, as few patients can access treatment close to home. This leads to prolonged time away from home for many, limited social and family support, and increased travel burdens [4]. Parents and caregivers may face isolation and long-distance travel, while also having to manage other responsibilities such as caring for other children, missing work, financial strain, and disruptions to the child’s education [10]. Length of stay (LOS) after MRT depends on factors including the radiopharmaceutical used, radioactive decay, disease burden, biological clearance, and treatment protocol, ranging from one day to four weeks [11, 12].

Planning and delivering MRT cancer treatment demands precise, individualised care. Adaptations are guided by the patient’s and family’s physical, psychological, social, environmental, spiritual, and treatment-related needs [1]. Regular multidisciplinary team (MDT) meetings support informed clinical decisions through shared expertise. Staff such as nurses or therapeutic radiographers specialising in MRT play a key role, serving as the main liaison between patients and services. They advocate for vulnerable patients, uphold safety, ensure continuity of care, and communicate patient needs within the MDT [13, 14].

Holistic Needs Assessments (HNA) have been associated with improved identification of patient concerns, and may support earlier intervention and more structured care planning by healthcare professionals, with potential benefits for patient outcomes and quality of life [15].

Although HNA are established in some oncology departments, their implementation varies across clinical practices. The lack of standardisation can lead to differences in the types of needs assessed or how they are addressed, making it difficult to compare and generalise findings. Reports on the use of HNA tools in MRT are limited and to date [16], no research has focused on the holistic care needs of parents or caregivers of children and young people receiving MRT. This gap may be due to the relatively small number of paediatric and teenage patients receiving this treatment compared with adult patients. This Clinical Service Review aimed to identify and quantify the needs of children and young people undergoing MRT, and their families.

## Methods

All parents and caregivers of patients aged under 18 years treated with molecular radiotherapy between 2018 and 2022 at one hospital were included. Caregivers completed an HNA form before admission (Appendix). Thematic and axial coding identified category patterns, followed by selective coding to define core themes and connections [17].

Written informed consent was given by all parents (or older patients if appropriate) before treatment. The United Kingdom Health Research Authority regards Clinical Service Review as not requiring Research Ethical Committee approval. Anonymised clinical data were compiled in accordance with data protection laws and this Clinical Service Review was approved by institutional governance processes without the need for ethical review.

## Results

### Patient sample

**Fig. 2:**
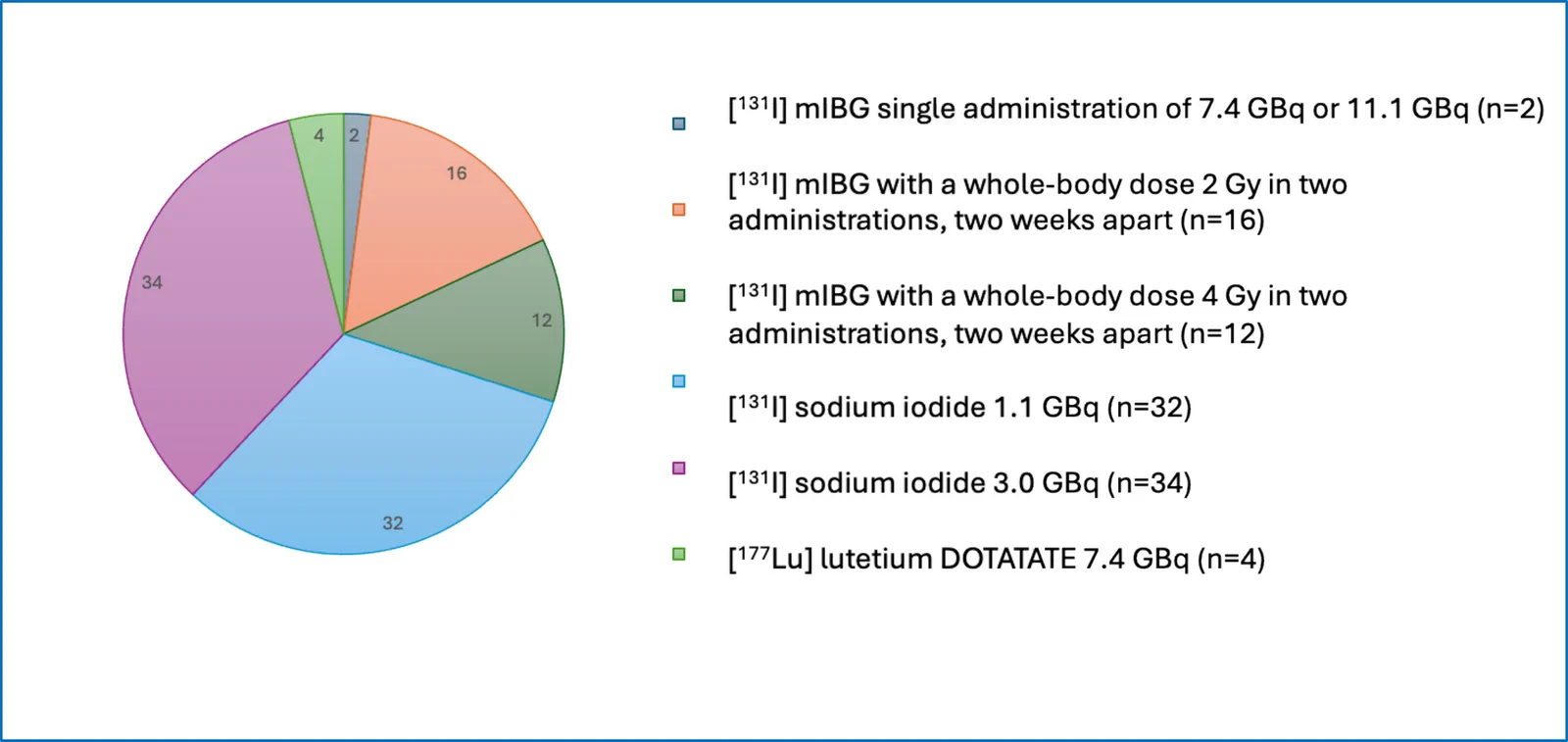
Molecular radiotherapy type

Between 2018 and 2022, 61 new referrals for children and teenagers treated with MRT were identified. A completed HNA was available for 50 of 61 patients (82%). The median age of the patients was 13 years, ranging from 3 to 17 years. The type of treatment received is detailed in Fig. 2*.*

### Main theme 1: strengths and supports

**Fig. 3:**
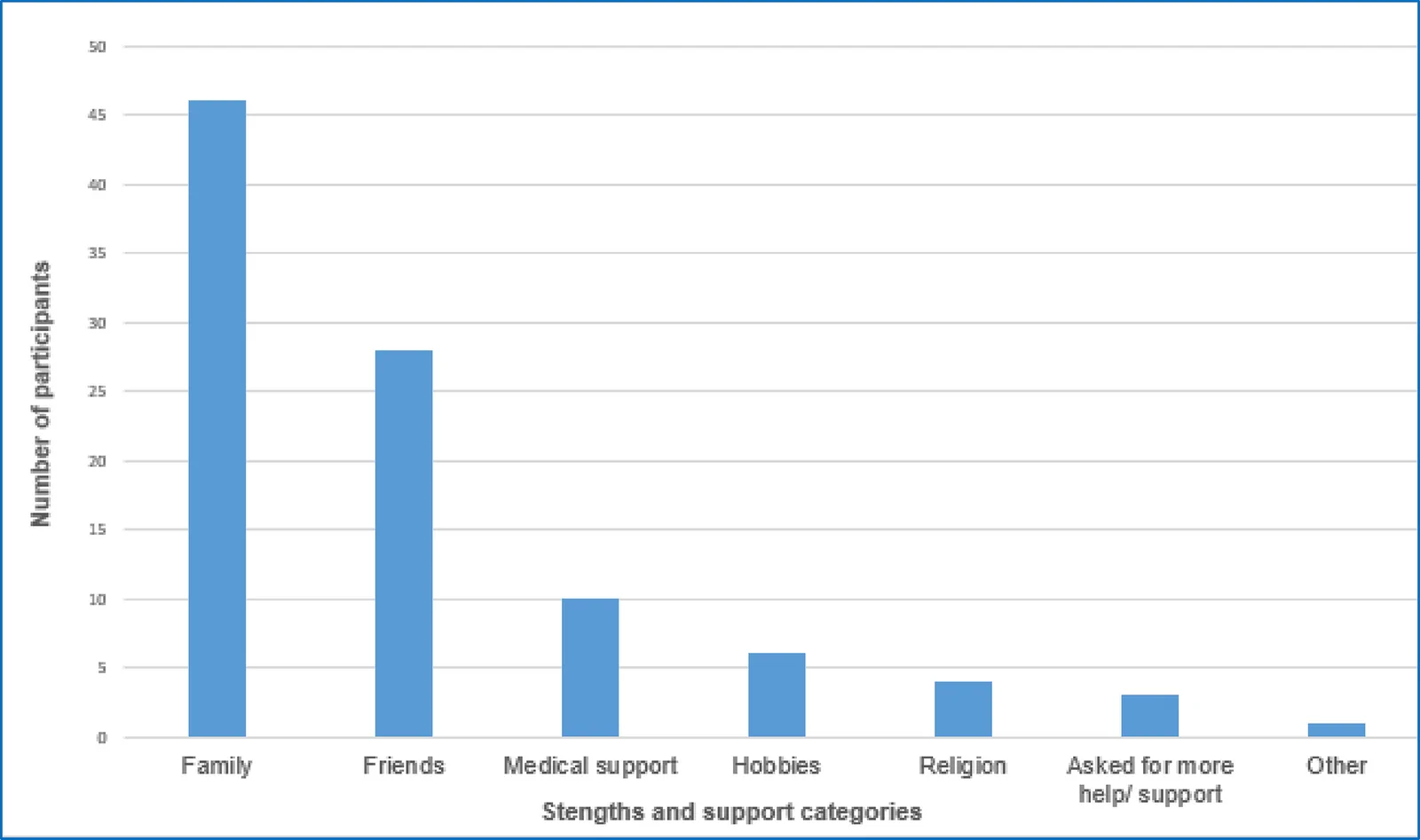
Participant’s strengths and support categories

Support was primarily drawn from immediate family (92%), followed by friends (56%) and medical professionals (20%). Additionally, 12% accessed counsellors or therapists. Smaller proportions found support through hobbies (12%) and religion (8%), while 6% requested more help. Participants emphasised the value of open family communication—“sharing feelings,” “honest discussions,” and “talking it through allows me to move forward.” They also noted the need for “distractions,” “taking my mind off things,” and “positive encouragement” to cope (Fig. 3)*.*

### Main theme 2: current stressors

The primary concern reported by participants was financial stress related to MRT treatment, cited by 98%. The second most common issue was separation from family (52%), including childcare and household responsibilities during treatment. Cancer-related anxieties—such as fear of recurrence, treatment effectiveness, and the potential loss of a child—accounted for 24%. Isolation was reported by 36%, while 22% mentioned concerns related to schooling and education.

Overall, 51% had financial concerns mainly linked to lost income due to time off work. A comparison of MRT type, length of stay (LOS), and financial impact revealed a strong correlation between longer hospital stays and financial strain. Patients on the complex double administration [^131^I]mIBG protocols, involving up to five weeks of inpatient care, represented 75–83% of those reporting such concerns. Additional stressors included unemployment (14%), travel, accommodation, and parking costs (20%).

Participants described feeling “stressed,” “scared of the future,” and overwhelmed by constant financial worry. Many had to take career breaks or leave jobs to provide care. Logistical challenges and separation from family were also significant, with comments like, “It is scary to come away from home, who will look after my family?” reflecting these burdens.

### Main theme 3: mental health status

**Fig. 4:**
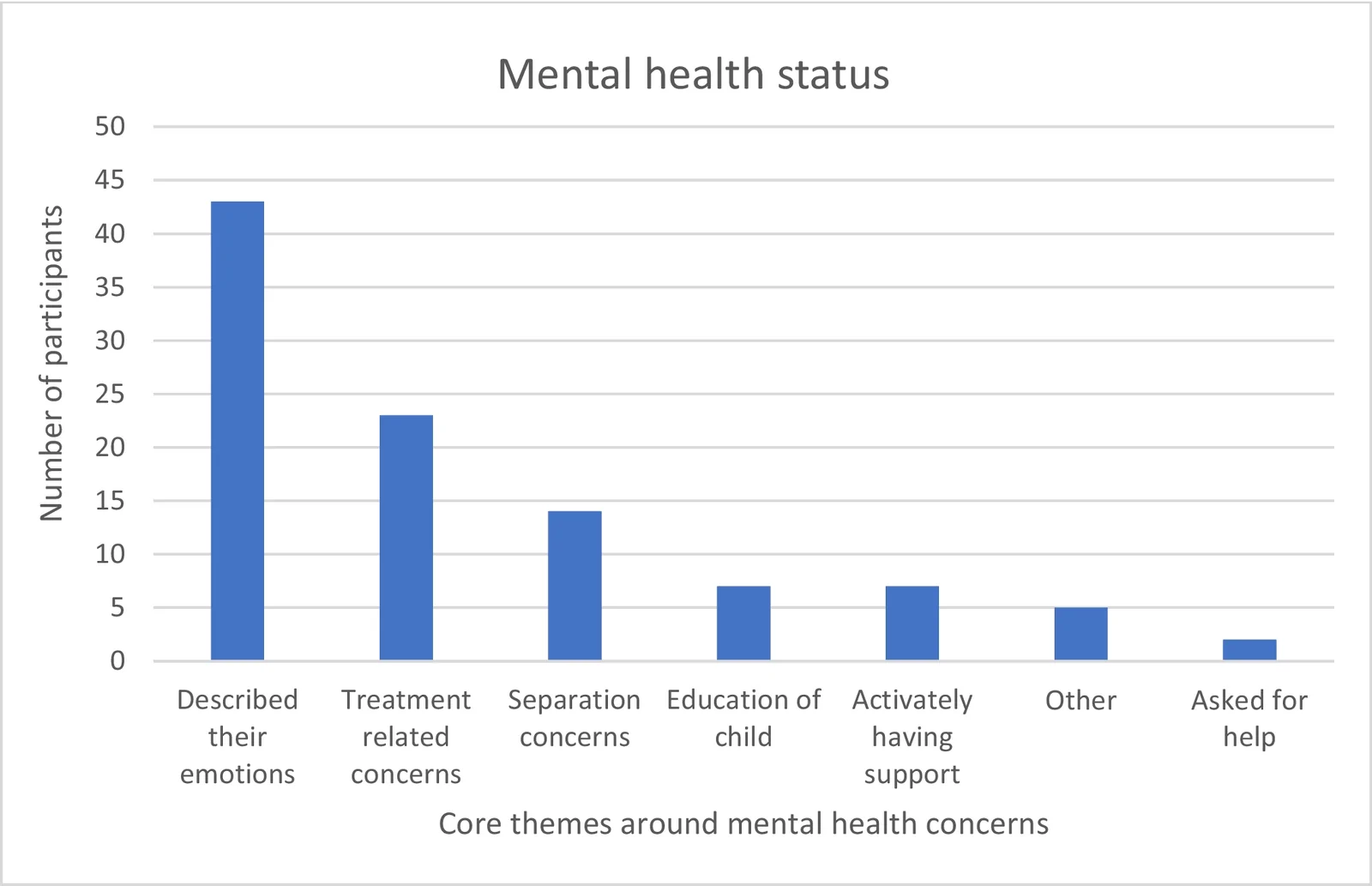
The core themes around mental health concerns

Negative emotions reported included feeling “*helpless, anxious, depressed, struggling, overwhelmed*,” and “*frustrated.”* Participants described the experience as a “*very stressful time for us all*,” with one noting, “*my child may cope, but I will need family support.*” The most common treatment-related concerns were isolation (16%) and work absence (8%), consistent with earlier HNA findings (Fig. 4). Separation from support networks, including family and siblings, was the third most frequent concern, with worries about childcare and siblings’ mental health both at 12%.

### Main theme 4: treatment-related concerns

34% percent of participants responded to whether they received enough information. Of these, 50% expressed concerns about their child’s radioactivity during MRT, including isolation (24%), boredom (18%), and fear of radiation (6%). Isolation worries were more common among caregivers of children receiving [^131^I]mIBG with two administrations, two weeks apart with an admission time of 4 to 5 weeks with 50–67% reporting concern, compared to 24% for those undergoing shorter radio-iodine treatments. This suggests that longer hospital stays increase anxiety about isolation. Participants described feeling “worried about being confined and claustrophobic” and emphasised the need for “activities,” “play support,” and “attention.”

Medical concerns about recurrence and remission accounted for 16%, while 30% fell into an “other” category, including issues like education (children missing school because of time in hospital), requests for help (including practical support with parking, provision of a prayer mat, entertainment and lack of cooking facilities), and radiopharmaceutical supply concerns (due to failure by commercial suppliers).

### Main theme 5: religious or spiritual beliefs

**Fig. 5:**
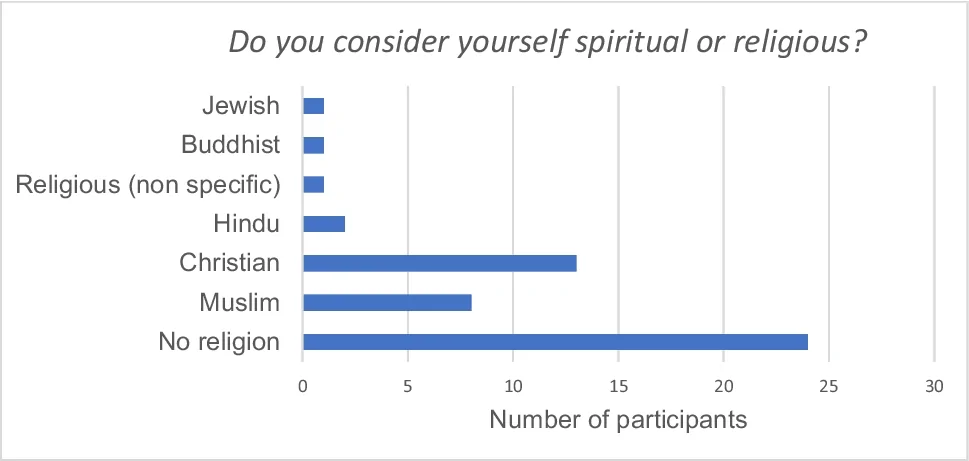
Participant’s religious and spiritual beliefs

Of the participants, 52% identified with a religion or spiritual belief, primarily Christian or Muslim (Fig. 5). The remaining 48% reported no religious or spiritual affiliation. Among them, 16% highlighted the importance of prayer, with statements such as “praying strengthens our body and soul” and “spiritual belief helped immensely in coping with the current situation.” Additionally, 6% expressed that they were losing or questioning their faith.

### Main theme 6: additional comments

22% of participants provided additional comments, highlighting concerns about the child’s education (4%), logistical issues like parking and cooking facilities (4%), the need for play specialist support (2%). Entertainment facilities inside the room, supply issues of the radiopharmaceutical, use of a prayer mat and struggling with the unknown were also mentioned.

## Discussion

We have prospectively mapped the anxieties and concerns reported by parents and caregivers of 50 children and young people receiving MRT using the HNA form. We did not identify any variation in relation to patient age or sex, but worries increased with longer LOS.

Most parents or carers rely on immediate family for support (92%), consistent with research linking social support to better outcomes and less stress [18]. HNA showed emotional support needs, desire for open communication, and adequate medical information, but less psychosocial care [19, 20]. Financial burden was the main stressor for nearly all families, reflecting national data on income loss and work disruption in cancer caregiving [21]. Hospital LOS worsened financial strain due to travel and other costs, especially given the treatment centre’s location, often far away from home [22–24].

Only 20% reported support from medical professionals, possibly because care was coordinated locally before treatment. Studies have advocated that carers or parents may not seek support when they need it, as they put the child’s needs before themselves [25]. Integrated multidisciplinary care is established for families at the referring principal treatment centre from the time of original diagnosis, and the parents of carers may not see the importance of reiterating information about care already provided to the team at the, often remote, MRT facility. Careful handover of relevant information between the referring and treatment centres is critically important, and this is an iterative conversation involving medical staff and key-workers, rather than a one-off communication. It remains crucial for professionals to continually offer diverse support throughout the cancer journey.

Parents or carers often experience fear, exhaustion, and negative emotions during treatment, influenced by treatment intent and child’s quality of life [25, 26]. It is evident that parents worry about their child missing out, boredom, missing their loved ones and concerns about the impact of treatment or time away has on their personal development. Peer relationships and academic development are unquestionably affected by being physically isolated not only during MRT treatment but also after completion with potential radiation restrictions that can prohibit close prolonged contact with others [26, 27]. It is therefore not surprising that the increased LOS also increased carer anxieties around the time within isolation.

Despite National Health Service (NHS) England’s Improving Access to Psychological Therapies programme, many carers face long waits for mental health support [28, 29]. Psychological care must be routinely offered, but current delays worsen vulnerability. Addressing the identified needs requires additional resources that are not readily available in healthcare, so gaps in care still exist [30].

Separation and isolation during MRT cause anxiety and depression, mirroring effects seen in COVID-19 lockdowns. Isolation impacts children’s peer relationships and education, with longer LOS increasing caregiver anxiety.

Teenagers may experience greater stress due to understanding their condition more deeply, affecting family dynamics [1]. Caregivers often focus on the child’s needs, requesting play specialist support during isolation. Virtual platforms can help maintain social and educational connections, supported by post-pandemic developments in remote learning and peer mentoring. Schools are legally required to arrange education during hospital stays; however, in the context of MRT patients this may not be face to face due to the stringent radiation protection guidelines. Input from schools in advance admission is vital.

Psychological care for carers of cancer patients is under-resourced, with over 75% receiving no support [30]. While waiting for formal care, alternative options like meditation, complementary therapies, or peer support should be explored. Health professionals often lack training and knowledge of local services, highlighting the need for a national resource for efficient signposting [31, 32]. Online peer support groups within MRT services could reduce anxiety and isolation, offering practical help while respecting radiation precautions. The impact of COVID-19 lockdown on coping with MRT isolation warrants further study [31].

Several studies link cancer patients’ spirituality and religion to better well-being, offering hope, identity, and comfort during treatment. Religion is community-based, while spirituality is personal [33], explaining why 48% of participants identified as non-religious despite having faith. The Office for National Statistics (ONS) recommends voluntary religion questions with a “prefer not to say” option placed last to reduce bias [34]. This study had a 54% non-response rate, much higher than national surveys, likely due to fear of prejudice. Ignoring spiritual needs can reduce patient satisfaction and quality of life; ethical guidelines urge health professionals to address spirituality in care [35].

The HNA helps to identify & start conversations around complex issues but this is only the first step.

While the holistic needs assessment raises awareness of issues, to help both staff and patients, together with reducing barriers and fostering engagement and relationship building, it does not cover all areas of support. For example, social services input, specific issues such as nutritional advice, physiotherapy or occupational therapy input if required and the bigger family picture, such as potential psychological support for siblings or other family members.

Without follow-up and the allocation of resources to address these needs, the tool can become ineffective. Healthcare staff need training in sensitive conversations and coaching to improve HNA use and support, especially around financial and social issues [1, 9]. Clear roles and planning are essential for timely, effective care. Signposting to charities or groups that can support with benefits needs to be explored prior to hospital stays.

Longer hospital stays increase financial and isolation concerns, emphasising the need for flexible working supported by employers and social policies [36]. Additional resources like play specialists and charities (e.g., Spread a Smile) can provide respite and support [37].

### Limitations of the study

This study has limitations, including 18% of missing data in the paper or electronic records. We attribute this to families failing to complete the assessment as requested, because completion of the HNA was not considered mandatory during the study period. However, the large five-year sample allowed meaningful analysis. As non-completion of the HNA affects the support which we can give families, future practice should routinely use this tool for all patients as per national guidelines [1].

## Conclusion

The study highlights key holistic care needs of parents and caregivers of children or teenagers receiving MRT, emphasising that support must extend beyond the patient. Despite some feeling adequately informed, many needs remain unmet, especially psychosocial support, financial strain, and treatment-related concerns like isolation and separation. Longer hospital stays increase these challenges, underscoring the need for flexible work arrangements and practical solutions from health professionals. Collaborative MDT planning and use of HNA tools are crucial for personalised family care. Understanding these issues can guide improvements, such as virtual support, education during treatment, and better use of local services, which should be integrated into ongoing professional development.

## Supplementary Information

Below is the link to the electronic supplementary material.

ESM1 Appendix A. Holistic needs assessment (DOCX 27.1 KB)

## Data Availability

The pooled data on which this paper is based was collected on forms completed by patients or their parents or carers. All authors were members of the clinical care team. To protect patient confidentiality, individual data will not be made available.

## Abbreviations

HNA: Holistic needs assessment
IRR17: Ionising Radiation Regulations 2017
LOS: Length of stay
MDT: Multidisciplinary team
mIBG: Meta iodobenzylguanidine
MRT: Molecular radiotherapy
NHS: National Health Service
ONS: Office for National Statistics
