# Supplementary material for: Exploring holistic care needs in paediatric and teenage molecular radiotherapy: a parent and carer perspective
Source: Support Care Cancer. 2026 Sep 22;34(10):1000. doi: 10.1007/s00520-026-11214-8 (PMC13597573; doi:10.1007/s00520-026-11214-8)
Supplement: Supplementary file 1 [file 520_2026_11214_MOESM1_ESM.docx]

**Appendix A: Holistic Needs Assessment**

Paediatric Oncology Assessment Measure for Molecular Radiotherapy: Parent/Carer

| Office Use Only | | |  | Information Provided | | |
| --- | --- | --- | --- | --- | --- | --- |
|  | |  |  |  | |  |
| Patient Name |  | |  | **Goal of Assessment** |  | |
|  |  | |  |  |  | |
| Date of Administration |  | |  | **Confidentiality** |  | |
|  |  | |  |  |  | |
| Details of Lead Clinician |  | |  | **Care Plan Development** |  | |
|  | | |  | **Support Organisations** |  | |

| **Topics** | **Notes** | **Alerts**  **(Risk or Protective)** | **PIC** |
| --- | --- | --- | --- |
| *Tell us about your child and your family’s story* | **Recent History** | *(For office only)* | *(For office only)* |
| **Religious or spiritual beliefs**  *1. Do you consider yourself spiritual or religious? Do you have spiritual beliefs that help you cope with stressful experiences? What importance does faith or belief have in your life?*      *2. Are you part of a spiritual or religious community?* | | *(For office only)* | *(For office only)* |
| **Mental health status**  *1. How are you coping with things at the moment?*      *2. How do you think you and your child will cope with being in isolation during treatment at UCLH?*      *3. Are there any family members at home you are worried wont cope while you are away? If yes what could we do to help?* | | *(For office only)* | *(For office only)* |
| **Current stressors**   1. *What are you most worried about, within or outside this cancer experience?*        2. *Will admission to UCLH result in any financial issues/issues with work that we could help with?*        3. *Are there any important upcoming family events we need to be aware of?*        4. *Have alternative care plans been made for other children/family members while your child is in hospital?* | | *(For office only)* | *(For office only)* |
| **Strengths and supports**   1. *Who helps you when you are worried? Who do you talk to?*        2. *How do you get through bad times? What are the things that you have in your life, or within yourself, that are going to help you get through this experience?* | | *(For office only)* | *(For office only)* |
| **Treatment concerns**  *Do you feel you have been provided with enough information about the treatment, including side effects, from your primary hospital?*      *If no, what additional information would be helpful?*      *Have you been made aware of the reasons that might mean your child will be unable to start treatment or that treatment may need to be stopped?* | | *(For office only)* | *(For office only)* |
| **Additional Comments**  *Is there anything the team could put into place before your child is admitted that might help?* | | *(For office only)* | *(For office only)* |
| **Areas to Address**      *(For office only)*  Clinician’s Signature:  Date:  /   / | | | |
| I,       (Patient’s name/Patient’s Guardian), understand that the above information will be used by my treating team to further develop my care plan. | | | |
| Patient’s Signature:  Date:  /   / | | | |
